# A System for Retrofitting Conventional MRI Systems for Simultaneous Multinuclear MRI/MRS

**DOI:** 10.1002/nbm.70326

**Published:** 2026-06-18

**Authors:** Jue Hou, Courtney Bauer, Edith Touchet‐Valle, Mary P. McDougall, Steven M. Wright

**Affiliations:** ^1^ Department of Electrical and Computer Engineering Texas A&M University College Station Texas USA; ^2^ Department of Biomedical Engineering Texas A&M University College Station Texas USA

**Keywords:** frequency translation, MR spectroscopy, receiver array, RF mixers, simultaneous multinuclear spectroscopyX‐nuclei

## Abstract

Proton magnetic resonance imaging (MRI) and spectroscopy (MRS) are widely used in clinical and research applications. Recent interest in X‐nuclei studies highlights their ability to provide additional biochemical information, but the intrinsically low X‐nuclear signal‐to‐noise ratio (SNR) significantly increases scan time. Simultaneous (rather than serial) acquisition of multiple nuclei can significantly reduce experiment time, but most conventional MR systems lack this capability without modifications. We present a cost‐effective system that enables simultaneous multinuclear imaging and spectroscopy on conventional MR spectrometers. Our approach offers enhanced flexibility for multinuclear experiments, supports multinuclear array receive capability, and maintains phase stability in the radio frequency (RF) chain. The proposed system comprised multiple transmit and receive mixing channels and a four‐channel flexible local oscillator (LO) source. By interfacing with the spectrometer, simultaneous transmit and receive at different frequencies were achieved. The performance of the system was evaluated through bench measurement and phantom multinuclear MRI and MRS experiments. Transmit and receive channel isolation of better than 30 dB was measured on the bench. Simultaneous excitation and reception of ^2^H and ^23^Na gradient echo images were acquired, as well as interleaved excitation with simultaneous reception of ^1^H, ^2^H, and ^23^Na FIDs. Water‐suppressed ^1^H and ^31^P MRS were performed simultaneously on phantoms mimicking muscle metabolites. Results across all experiments showed no signal‐to‐noise ratio (SNR) loss compared to single‐frequency operation. The proposed system supports multiple variations of simultaneous experiments on conventional MRI systems, demonstrating its flexibility in configuring experiments with varying numbers of nuclei (2–4), different transmit modes (simultaneous or interleaved), and supporting receive array coils of up to 16 channels, while maintaining phase stability in the RF chain without the need for retrospective correction.

## Graphical abstract

We present a low‐cost system for retrofitting conventional narrow‐band MRI spectrometers with simultaneous multinuclear capability using heterodyne frequency translation. The system supports up to four nuclei and 16 receive channels, with reconfigurable transmit modes and inherent phase stability through a shared local oscillator architecture. Simultaneous and interleaved multinuclear acquisition of ^1^H, ^2^H, ^23^Na, and ^31^P was successfully demonstrated at 4.7 T with no SNR penalty compared to single‐frequency operation, at a total component cost under $15,000.

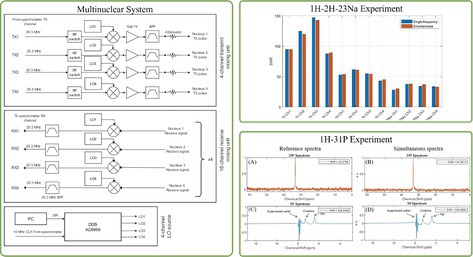

## 1 Introduction

Magnetic resonance imaging (MRI) and magnetic resonance spectroscopy (MRS) are widely used diagnostic tools used in both research and clinical applications. The majority of those studies are done with ^1^H due to its high abundance and high sensitivity. However, nuclei other than ^1^H can also emit nuclear magnetic resonance (NMR) signal, such as ^2^H, ^23^Na, ^31^P, ^13^C, and ^17^O. They are usually referred to as X‐nuclei and are of great interest due to the complementary biochemical information they can provide [1, 2, 3, 4, 5].

In X‐nuclear studies, usually two or more nuclei are investigated in the same experiment. In most cases, ^1^H imaging is needed to provide anatomical information and to perform B_0_ shimming. Although most studies focus on scanning one X‐nucleus, studying multiple X‐nuclear biomarkers for a given disease is of increasing interest [6, 7, 8]. However, X‐nuclei are less sensitive and require longer scan times to accommodate the necessary increase in averages. Furthermore, each nucleus is typically scanned sequentially as most of the MR systems only support single frequency operation. Sequential scanning obviously increases the overall time required for the study, in some cases by an amount that prohibits practical clinical application. To overcome this issue, researchers have investigated the utility of interleaved or simultaneous multinuclear experiments, terminologies that have been proposed and defined by a review paper on this topic [9]. While reference [9] defines these terms based on how the signal is received, in this paper, we use them more broadly to describe both transmit and receive: “simultaneous” refers to the case where radio frequency (RF) pulses and/or signals at multiple frequencies are transmitted and/or received at the same time, while “interleaved” refers to the case where different nuclei are excited and/or received sequentially within the same TR. These concepts were introduced and explored in the earliest days of NMR. For example, a lab‐built spectrometer was developed for interleaved excitation and acquisition of ^31^P and ^13^C spectra [10]. By using two separate spectrometers, ^1^H and ^31^P NMR measurements were performed simultaneously in rabbit skeletal muscle [11]. Similarly, ^1^H imaging and ^31^P localized spectroscopy were also simultaneously acquired with two spectrometers [12]. Finally, by switching the system operating frequency, interleaved ^1^H and ^23^Na imaging were acquired in vivo [13].

In the past decade, X‐nuclear studies have seen a rapid growth with recent advances in data acquisition techniques and MR hardware. With the advancement of hyperpolarization methods and the increased accessibility to ultra‐high field magnets, it is more feasible to acquire X‐nuclear signal in a reasonable scan time [14, 15]. Magnetic resonance spectroscopic imaging (MRSI) with X‐nuclei has also been demonstrated in a clinical setup [3, 16]. The introduction of novel double‐tuned and multituned RF coils has simplified experiment setups and made it possible to simultaneously transmit and receive at multiple frequencies [17, 18, 19]. Especially with the development of multituned receive arrays, higher X‐nuclear sensitivity and acceleration can be achieved [20, 21, 22, 23, 24, 25].

With the aforementioned advances, there has been a renewed interest in performing simultaneous or interleaved multinuclear studies on modern systems. Simultaneous and interleaved ^1^H and ^31^P acquisition were demonstrated on a clinical 7‐T system and later applied to in vivo leg muscle studies [26, 27]. Interleaving a deuterium metabolic imaging (DMI) scan with conventional imaging was shown to yield additional ^2^H labeled metabolic information without extending the scan time [28, 29]. By adjusting the receiver frequency, ^1^H magnetic resonance fingerprinting (MRF) and ^23^Na MRI were simultaneously acquired on a 7‐T clinical scanner [30]. Other applications include simultaneous ^1^H/^19^F imaging for motion correction [31] and interleaved ^1^H navigators for ^23^Na motion correction [32].

Unfortunately, one major challenge is the lack of support for simultaneous or interleaved multinuclear experiments for most conventional human MR systems. Although some can support X‐nuclear operation, the RF bandwidth is intrinsically narrow and not able to cover the bandwidth across multiple X‐nuclei at the same time. Therefore, modifications including additional RF channels, broadband amplifiers, external frequency synthesizers, and custom pulse sequence programming are usually required, and the modifications can vary greatly depending on the specific MR system configuration, multinuclear coil setup, and the desired experiment type. In addition, a common approach in these studies is to inject an external LO signal into the receiver chain to shift the reception frequency to the desired nucleus. However, since this external LO is not synchronized with the transmit chain, any phase noise or drift in the LO is not common to both the transmit and receive paths and therefore does not cancel out, introducing a phase shift in the received signal that requires additional postprocessing to correct [26, 27, 28, 29, 30]. Additionally, the proton‐only systems would need a significant vendor‐specific X‐nuclear upgrade, which can cost up to $0.5 M [33] before any of the above could be considered, which greatly discourages the exploration of simultaneous multinuclear possibilities for many researchers. The frequency translation approach using heterodyne mixing has also been demonstrated for X‐nuclear phased‐array MRI on clinical systems [34], for multichannel 13C spectroscopy [35], and as a portable frequency converter for X‐nuclear MRS and MRI on proton‐only scanners [36]. However, these systems are designed for single‐nucleus operation at a time and do not support simultaneous or interleaved multinuclear acquisition.

In this paper, we present a system for retrofitting conventional narrow‐band spectrometers with simultaneous and/or interleaved multinuclear capability. The proposed system consists of a multichannel local oscillator (LO) source and multiple reconfigurable transmit and receive mixing channels. As configured here, the system can support array receive coils of up to four channels for up to four different nuclei in one experiment. The number of channels readily could be scaled up. The system is also reconfigurable to support different multinuclear transmit methods (simultaneous or interleaved) and different frequencies based on the needs of the study. Since the proposed design utilizes the heterodyne receiver structure where both transmit and receive frequencies are converted by the same LO, any extra phase introduced by the LO is common to both paths and cancels out, ensuring phase stability of the received signal and eliminating the need for retrospective phase correction. Finally, the proposed system is built using affordable and commercially available components, making it a low‐cost and scalable approach accessible to engineers and researchers.

The system performance is demonstrated through bench measurements and MR experiments. First, the RF isolation for different transmit and receive channels is measured on the bench. Next, a three‐frequency interleaved FID experiment and a two‐frequency simultaneous gradient echo (GRE) experiment are conducted solely for hardware performance evaluation without biological relevance.

Finally, our group has studied thawed cryopreserved ex vivo golden retriever with muscular dystrophy (GRMD) skeletal muscle samples using ^1^H and ^31^P spectroscopy. This is a particularly relevant application as a challenge with cryopreservation is the acceleration of biochemical degradation at room temperature [37], another potential motivation for simultaneous acquisition and decreased overall study time. The proposed system is configured to demonstrate its ability to support such a simultaneous ^1^H and ^31^P study.

## 2 Methods

### 2.1 The Proposed System and Implementation

#### 2.1.1 The Proposed Multinuclear System

**FIGURE 1:**
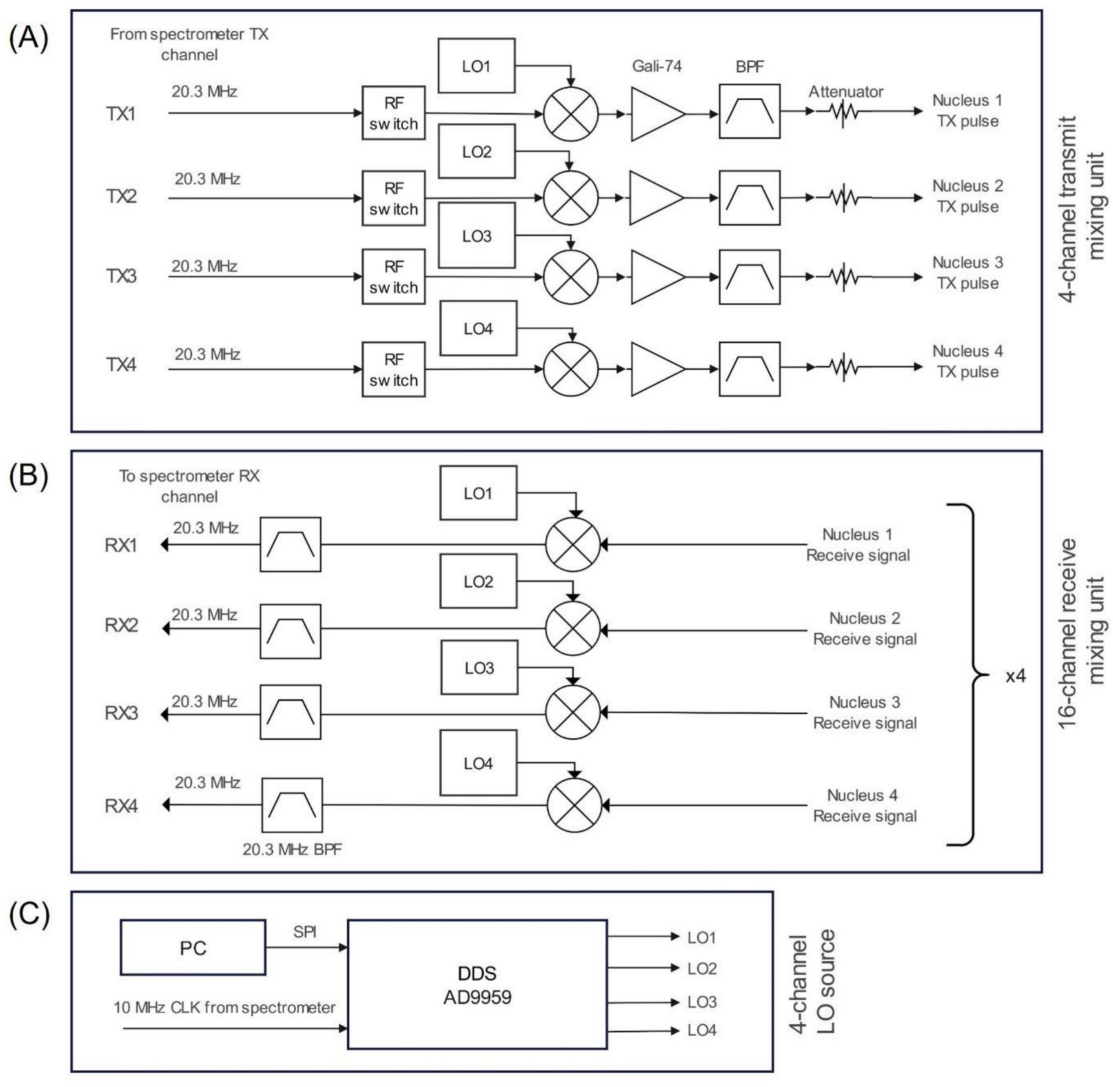
The functional diagram of the add‐on system. (A) The transmit mixing unit contains four independent mixing channels. The RF switches are used to enable or disable each channel and controlled by the TX gate from spectrometer. The step attenuators are used to adjust the transmit gain of each channel independently. (B) The receive mixing unit contains 16 mixing channels, and every four channels share the same LO signal. (C) The four‐channel LO source is based on the ad9959 DDS and controlled by a PC to set the frequencies. Each LO output is split and fed to both the corresponding mixing channels for both transmit and mixing unit. Abbreviations: BPF, band‐pass filter; CLK, clock; DDS, direct digital synthesizer; Gali‐74, Gali‐74 low‐noise amplifier (Mini‐Circuits); LO, local oscillator; RF, radio frequency; RX, receive; TX, transmit.

The proposed system consisted of three major parts: a four‐channel LO source, a four‐channel reconfigurable transmit mixing unit, and a 16‐channel flexible receive mixing unit. The simplified system diagram is shown in Figure 1.

The transmit mixing unit included four transmit mixing channels and the corresponding filters and gain stages. Each channel was designed to convert the pulse‐modulated RF signal generated from the spectrometer to the desired Larmor frequency, preserving the pulse envelope while shifting the carrier frequency. At the input of each transmit mixing channel, an active RF switch was used to enable or disable that channel by connecting to the spectrometer TX gates. This allowed flexible selection of the number of nuclei of interest and the transmit method (simultaneous or interleaved) in an experiment. The frequency conversion was done by using a passive RF mixer ADE‐1L+ (Mini‐Circuits, Brooklyn, NY, USA). To compensate for the conversion loss due to the mixing, a low‐noise amplifier Gali‐74 (Mini‐Circuits) was added after the mixer. Then, a band‐pass filter centered around the desired Larmor frequency was added to select the proper mixing product. By changing the LO frequency that was provided to the transmit mixing channel, as well as the band‐pass filter, the transmit frequency could be adjusted. Before sending the transmit pulse to the RF power amplifier (RFPA), a step attenuator based on the HMC472 chip (Analog Devices, Norwood, MA, USA) was installed after the band‐pass filter for RF calibration. The attenuator has a step size of 0.5 dB and a maximum attenuation of 31.5 dB, which we have found to provide sufficient flip angle accuracy and repeatability for X‐nuclear experiments in our lab setup.

The 16 receive mixing channels were built based on an active mixer, ADL5801 (Analog Devices), and had been previously reported by our group [35]. By feeding different LO signals to different channels, the multinuclear signal was mixed down to the same intermediate frequency (IF) and received by the spectrometer. The 16 channels were divided into four sets, with each set sharing a common LO signal to support multinuclear signal reception of up to four frequencies at the same time.

A four‐channel direct digital synthesizer (DDS) AD9959 (Analog Devices) was used as the LO source for the transmit and receive mixers and was phase locked to the scanner's 10 MHz reference clock. It could provide four different LO frequencies and support up to four‐nuclei simultaneous experiments. The DDS was controlled via the serial peripheral interface (SPI) using an Arduino UNO microcontroller and could be easily programmed using a standard PC. The other key components used in the transmit and receive mixing units, along with their part numbers and manufacturers, are summarized in Table 1.

**TABLE 1:** Key components used in the transmit and receive mixing units of the proposed multinuclear system.

Component	Function	Part number	Manufacturer
20.3‐MHz BPF	IF filter	SXBP‐20R5+	Mini‐Circuits
30‐MHz BPF	^2^H TX filter	BBP‐30+	Mini‐Circuits
53‐MHz BPF	^23^Na TX filter	BBP‐60+	Mini‐Circuits
81‐MHz BPF	^31^P TX filter	BBP‐70+	Mini‐Circuits
200‐MHz BPF	^1^H TX filter	BPF‐B199+	Mini‐Circuits
1‐to‐3 splitter	RX signal splitting	AD3PS‐1+	Mini‐Circuits
1‐to‐4 splitter	TX signal splitting	ZFSC‐4‐1 W‐S+	Mini‐Circuits
RF switch	TX channel select	HMC544	Analog Devices
Step attenuator	TX gain calibration	HMC472	Analog Devices

#### 2.1.2 EVO Spectrometer

An EVO spectrometer (MR Solutions, Guildford, UK) system installed on an Oxford 4.7‐T magnet with a 40‐cm bore (27 cm with gradient coil inserted) in the Magnetic Resonance System Lab at Texas A&M University was used for the study. The spectrometer consists of 16 receiver channels to allow parallel signal reception and can support conventional single‐frequency operation up to 130 MHz. Therefore, the add‐on system was implemented not only to support simultaneous multinuclear operation but also to expand the spectrometer operation frequency to cover proton at 4.7 T.

#### 2.1.3 Integrating the Multinuclear System With the Spectrometer

**FIGURE 2:**
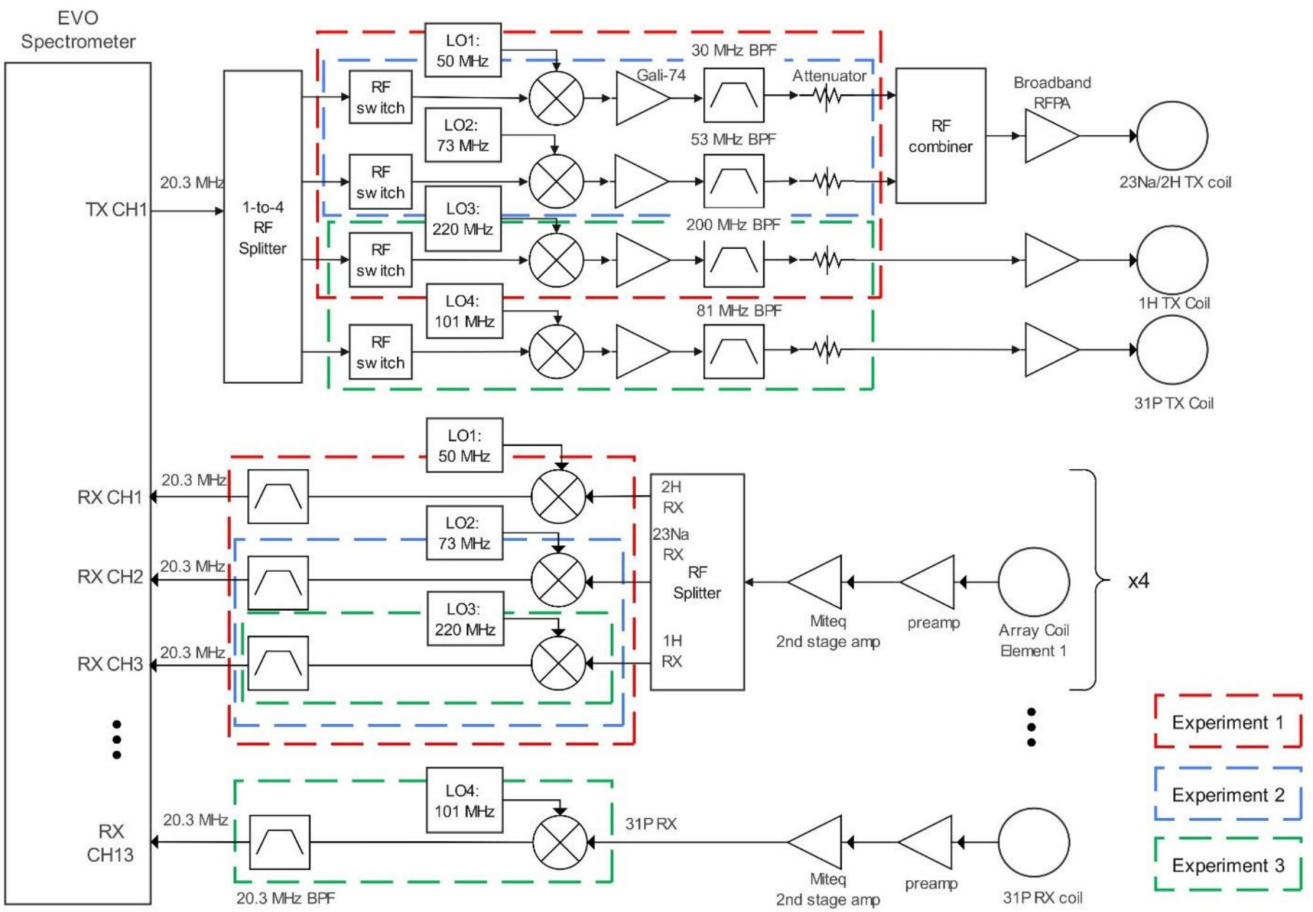
System integration diagram demonstrating the integration of the EVO spectrometer with the multifrequency system and the different coil setups. Although not implemented and tested at the same time, the connection for both coil setups is shown in one diagram for simplicity. The color‐coded boxes indicate the transmit and receive mixing channels used for three separate experiments. The TX gate signals from the spectrometer to the RF switches and the 10‐MHz line to the LO source are not shown here for simplicity.

**FIGURE 3:**
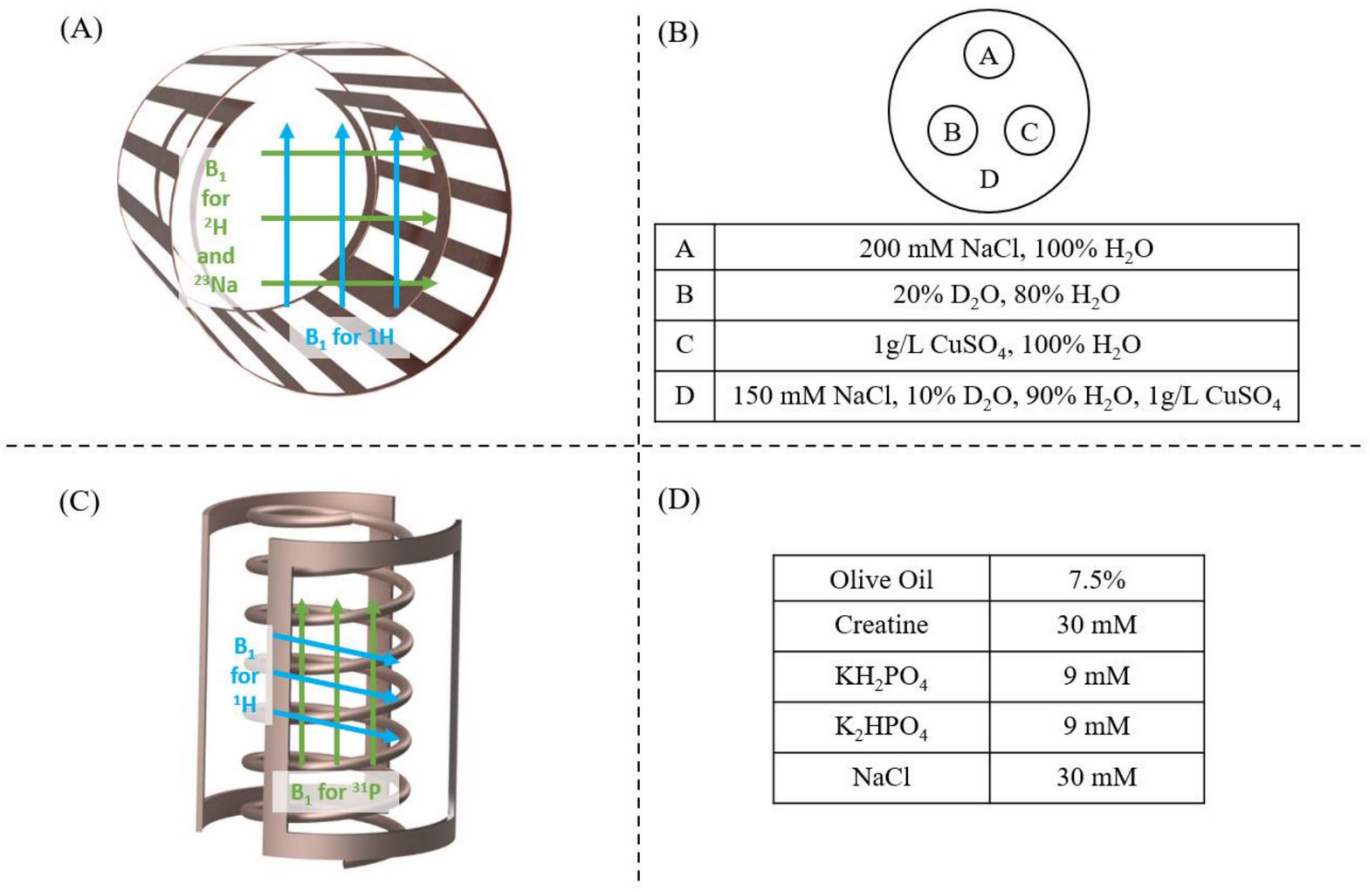
Illustrations and information of the RF resonators and phantoms utilized in this work. (A) CAD rendering of the triple‐frequency transmit resonator setup, consisting of a linearly polarized ^1^H birdcage resonator (outer) and a ^2^H/^23^Na saddle resonator (inner), demonstrating the orthogonal transmit field directions for geometric decoupling. The receive array and phantom are omitted for clarity. (B) Transverse cross‐section illustration of the ^1^H‐^2^H‐^23^Na phantom showing the structure and content of each sample within the phantom. (C) CAD rendering of the nested ^1^H saddle and ^31^P solenoid T/R resonators, illustrating the geometric decoupling between the two. (D) Content and concentration information of the ^1^H‐^31^P metabolite phantom, with individual samples labeled.

The spectrometer was set to operate at 20.3 MHz for both transmit and receive. This frequency was chosen due to the availability of narrow‐band 20‐MHz commercial band‐pass filters, and the additional 0.3 MHz was added to avoid interference with other equipment in the lab. On the transmit side, the transmit pulse from the spectrometer was split into the transmit mixers after a 1‐to‐4 RF power splitter to generate different transmit frequencies of interest, and the desired mixing channels could be selected by enabling the corresponding RF switches via TX gate signals. On the receive side, the received multinuclear signal was split into different receive mixing channels for down‐converting to 20.3 MHz for signal reception by the spectrometer receivers. The LO source was controlled by a standard PC and phase‐locked to the spectrometer 10 MHz clock line. Figure 2 illustrates the system integration with the EVO system and the different coil setups used in this work. To demonstrate flexibility, two coil setups were interfaced with the system. Setup 1 included a double‐tuned ^2^H/^23^Na transmit volume coil nested inside a ^1^H birdcage coil and a four‐channel triple‐frequency receive array. Setup 2 was a nested ^1^H and ^31^P transmit/receive (T/R) coil. The coils and experiments are explained with more details in later sections. Depending on the multituning method of the transmit and receive coils, additional RF power splitters could be implemented when interfacing with the system to combine or split the different frequency components, as illustrated in Figure 3.

### 2.2 Bench Measurement

The multinuclear system was evaluated on the bench to measure channel isolation for both the transmit and receive mixing units. It is crucial to avoid any in‐band or out‐of‐band signal coupling between different mixing channels on both transmit and receive sides. A vector network analyzer HP4195A (Hewlett‐Packard, Palo Alto, CA) was used to analyze the spectrum. All measurement data were recorded manually and plotted in MATLAB R2023b (MathWorks, Natick, MA).

For the transmit mixing channels, a 20.3‐MHz sinusoidal signal was inserted at the input of the 1‐to‐4 RF splitter. All the RF switches were set to the on position, and four different LO signals were provided to emulate the most demanding operation condition, in which transmit frequencies at ^1^H, ^2^H, ^23^Na and ^31^P were simultaneously generated. The conversion gain of all channels was set to 0 dB. At the output of each transmit mixing channel, the signal intensities at the desired frequency and the nondesired frequencies were measured.

For the receive mixing channels, a similar measurement was done for all 13 receive mixing channels which were used in this work. By providing four different LO frequencies, Channels 1–4 were set to receive ^1^H signal, Channels 5–8 were set to receive ^2^H signal, Channels 9–12 were set to receive ^23^Na signal, and Channel 13 was set to receive ^31^P signal. A sinusoidal signal at the targeted frequency of the mixing channel is inserted at the input, and the 20.3‐MHz output signal was measured at the outputs for Channels 1–13.

In addition, to verify that the transmit mixing process preserves the RF pulse envelope, the input and output waveforms of the transmit mixing channel were captured separately using an oscilloscope at the ^2^H frequency (30.7 MHz), both triggered off the same TX gate signal to ensure accurate timing comparison. A hard pulse was generated by the spectrometer at 20.3 MHz, and the output of the transmit mixing channel at 30.7 MHz was captured in a separate measurement. Since the conversion gain of the mixer is not unity, the gains were adjusted between the two measurements to allow direct visual comparison of the pulse envelopes. The rising and falling edges of both waveforms were examined to confirm that the pulse envelope shape was preserved after frequency translation.

### 2.3 MR Experiments

Three different simultaneous MR imaging and spectroscopy experiments were performed to demonstrate the frequency flexibility, phase stability, signal‐to‐noise ratio (SNR) performance, and adaptability to different multituned RF coils of the multinuclear system. Two sets of RF coil and phantom setups were used and interfaced with the proposed system. All the experiments were performed on the 4.7‐T Oxford magnet using the EVO spectrometer, and the connection of the spectrometer, the add‐on system, and the coil setups are shown in Figure 2. The hardware configuration parameters for the three experiments described in the following sections are summarized in Table 2.

**TABLE 2:** Hardware configuration parameters for the three MR experiments.

	Experiment 1: ^1^H/^2^H/^23^Na FID	Experiment 2:^2^H/^23^Na GRE	Experiment 3:^1^H/^31^P MRS
Nuclei	^1^H, ^2^H, ^23^Na	^2^H, ^23^Na	^1^H, ^31^P
TX mode	Interleaved	Simultaneous	Simultaneous
LO1 (MHz)	50	50	—
LO2 (MHz)	73	73	—
LO3 (MHz)	220	—	220
LO4 (MHz)	—	—	101
TX channels enabled	1, 2, 3	1,2	3,4
TX BPF (MHz)	200, 30, 53	30, 53	200, 81
RX channels used	1–12	1–8	9, 13

#### 2.3.1 RF Coil and Phantom Setups

The first setup consisted of nested birdcage and saddle transmit coils and a four‐element broadband receive array coil. The birdcage coil (D = 27 cm) was single‐tuned to the ^1^H frequency at 4.7 T and operates in linear mode. The saddle coil (L = 15 cm, D = 15 cm) was double‐tuned to the ^2^H and ^23^Na frequencies at 4.7 T. The two transmit coils have linear B_1_ field distributions near the center and were positioned with their fields orthogonal to one another to minimize coupling between the two, as shown in Figure 3A. The two coils both were actively tuned/detuned by two separate PIN diode drivers. During the experiment, one was detuned while the other was transmitting, and both were detuned during signal reception by the receive array. A four‐element circular array was placed inside the transmit coils. The array was designed to operate at ^2^H and ^23^Na frequencies and had been previously reported [21]. The array was also capable of receiving ^1^H signals, which made it suitable for demonstrating the three‐frequency simultaneous receive ability of the system. A multinuclear phantom was constructed for testing the proposed hardware. It was constructed with a cylindrical glass container with a diameter of 9 cm and height of 10 cm. It contained three different solutions in three small compartments, as well as a background solution in the main container. The phantom was filled with various concentrations of distilled water, D_2_O, NaCl and CuSO_4_ solution as illustrated in Figure 3B and provided sufficient signal for imaging at ^2^H and ^23^Na frequencies.

The second setup consisted of two single‐tuned T/R coils, a ^1^H saddle (D = 2 cm, L = 2.49 cm, α = 120°), and a ^31^P solenoid (D = 1.21 cm, L = 2.7 cm, *N* = 7) [38]. The two coils were nested together and geometrically decoupled, which allowed simultaneous transmit and receive at both frequencies, as shown in Figure 3C. These coil structures were designed to fit as closely as possible to 1 cm^3^ tissue samples. To demonstrate the utility of the proposed system, we performed testing on a phantom which contained inorganic phosphate and various ^1^H metabolites with physiologically relevant concentrations in skeletal muscle to allow optimization of low SNR ^31^P and water‐suppressed ^1^H spectroscopy experiment protocol. The sample content and concentrations are shown in Figure 3D.

#### 2.3.2 Three‐Frequency ^1^H, ^2^H, and ^23^Na FID Experiment

**FIGURE 4:**
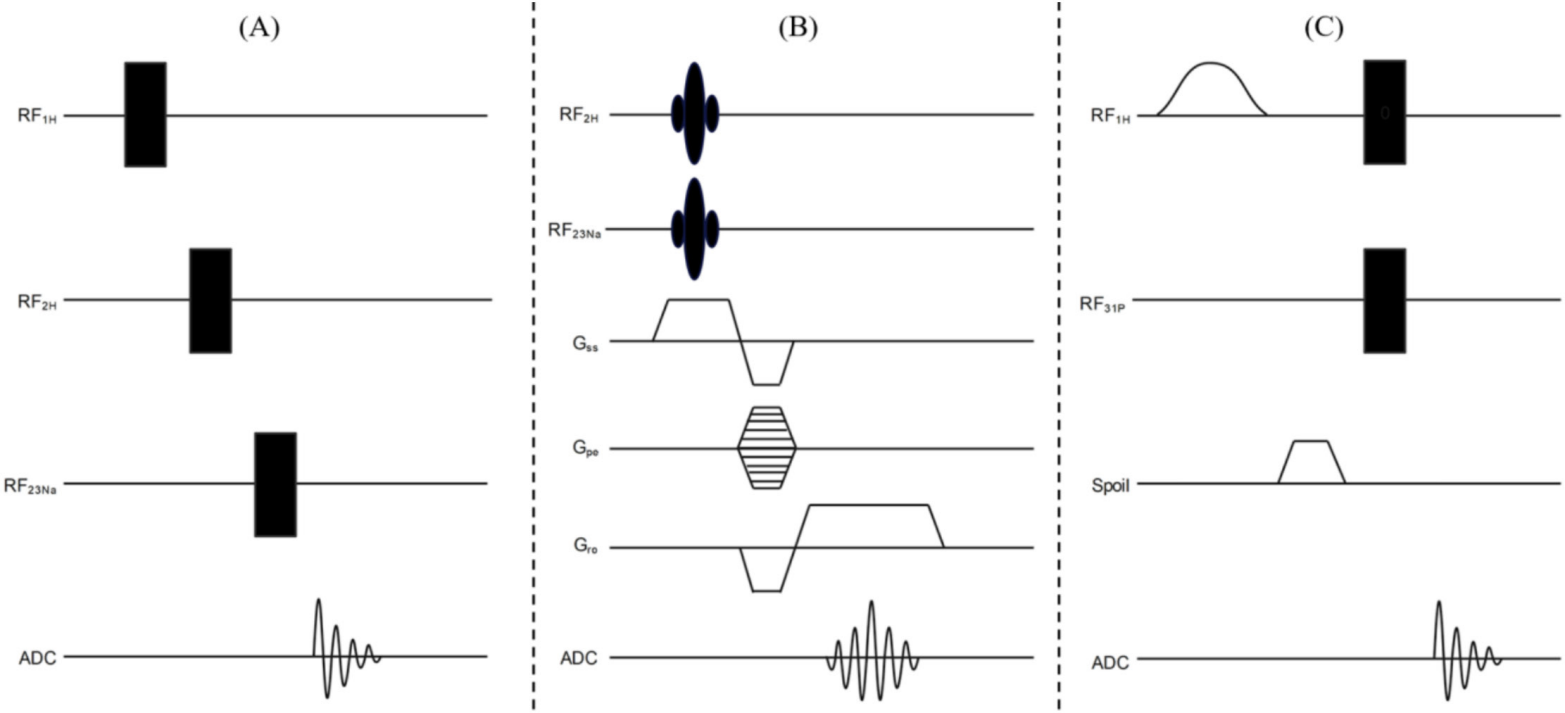
The pulse sequence diagrams for (A) the three‐frequency ^1^H, ^2^H, and ^23^Na FID experiment; (B) the two‐frequency simultaneous ^2^H and ^23^Na GRE imaging experiment; and (C) the two‐frequency water‐suppressed ^1^H and ^31^P spectroscopy experiment. The waveforms are for illustration purposes and not drawn to scale.

To demonstrate an interleaved transmit and simultaneous receive scenario, a three‐frequency FID sequence was modified based on a standard FID sequence and tested with the proposed system. In each TR, three hard pulses were programmed to transmit sequentially with independent amplitude and pulse width control. Three LO signals to mix between the 20.3 MHz and the Larmor frequencies were provided to transmit mixing Channels 1–3 and receive mixing Channels 1–12. Additional transmit gate signals were programmed into the sequence to trigger the RF switches in the transmit mixing channels and blank the RFPAs. The ^1^H, ^2^H, and ^23^Na band‐pass filters were inserted at the outputs of the corresponding transmit mixing channels. After the RF pulses, the receivers switch on and the MR signals at three different frequencies were digitized simultaneously. The three RF pulses were transmitted in the order of ^1^H, ^2^H, ^23^Na to maximize the ^23^Na signal, with pulse widths of 50, 150, and 300 μs respectively. The pulse amplitude was adjusted independently to achieve a 90° tip angle for ^23^Na, and small tip angles for ^1^H and ^2^H to reach relatively comparable signal strength across all three nuclei for hardware evaluation purposes, rather than to optimize SNR per nucleus. A 10‐μs instrumentation delay was added between the adjacent RF pulses to allow hardware such as RF switches, coil T/R switches, and RF amplifier blanking to function properly, and there was no additional delay added between the last RF pulse and the ADC (Analog‐to‐digital conversion). Other scan parameters were kept the same for all nuclei, as they were scanned simultaneously (TR = 3 s, 24 averages, SW = 25 kHz, 2048 samples). A simplified illustration of the pulse sequence is shown in Figure 4A. Reference spectra were acquired with single frequency operation of the system, and the ^1^H, ^2^H, and ^23^Na FIDs were collected individually. Then, the multinuclear system was configured to the three‐frequency interleaved excitation configuration by enabling all three transmit mixing channels in one experiment. The ^1^H, ^2^H, and ^23^Na RF pulses were transmitted sequentially, and the signals at the three different frequencies were acquired simultaneously. In order to compare the SNR in both cases, the RF pulse amplitude and the acquisition delay were kept the same for each nucleus.

#### 2.3.3 Two‐Frequency ^2^H and ^23^Na GRE Imaging Experiment

Second, ^2^H and ^23^Na GRE images were simultaneously acquired with the proposed system. Similarly, two LO signals to mix between the 20.3 MHz and the Larmor frequencies were provided to transmit mixing Channels 1 and 2 and receive mixing Channels 1–8. The ^2^H and ^23^Na band‐pass filters were inserted at the transmit mixing channel outputs as well. In this case, signals at two frequencies were transmitted and received simultaneously. In order to achieve this, a standard GRE pulse sequence was programmed with additional transmit gate signals to switch on the ^2^H and ^23^Na transmit mixing channels at the same time. The RF calibration for the two nuclei was done by independently adjusting the step attenuators for each transmit mixing channel. The scan parameters were set for the ^23^Na experiment (20‐mm slice thickness, 160‐mm field of view [FOV], 64 by 64 matrix size, 64 averages, TR/TE = 150/4 ms), and due to the simultaneous nature of this experiment, the same gradient strength was applied to the ^2^H experiment as well. The sequence diagram of this experiment was demonstrated in Figure 4B. Due to the different gyromagnetic ratios of the two nuclei, the ^2^H images had an effectively thicker slice and a larger field of view, which were zoomed in during post processing. For SNR comparison purposes, the multinuclear system was first configured to single frequency operation, and the ^2^H and ^23^Na images were acquired separately. Then the system was configured to two‐frequency simultaneous excitation by enabling both transmit mixing channels during excitation, and the ^2^H and ^23^Na images were acquired simultaneously.

#### 2.3.4 Two‐Frequency Water‐Suppressed ^1^H and ^31^P FID Experiment

To quantify ^1^H metabolites such as fat and creatine, it was necessary to suppress the water signal during the spectroscopy experiment and perform signal averaging. Due to the low SNR for the ^31^P signal in the muscle sample, significant averaging was also required. Similar to the experiment described in section 2.3.3, ^1^H and ^31^P LO frequencies were programmed. Additional transmit gate signals were programmed to switch on the two transmit mixing channels at the same time. The ^1^H and ^31^P bandpass filters were installed at the output of the two transmit mixing channels, and the RF calibration was done independently with the step attenuators for each channel. Other scan parameters were kept the same for both nuclei (TR = 5 s, 100 averages, SW = 5 kHz, 8000 samples). In order to perform water suppression, a narrow‐band Gaussian pulse centered around the water frequency was transmitted on the ^1^H channel only, followed by a spoiling gradient, which had no effect on the ^31^P frequency. Afterwards, the ^1^H and ^31^P RF pulses were applied simultaneously, followed by simultaneous acquisition of the signals at both frequencies. The experiment was done using both single frequency operation and simultaneous operation for comparison purposes. The pulse sequence used in this experiment is demonstrated in Figure 4C.

## 3 III Results

### 3.1 Bench Measurement Results

#### 3.1.1 Transmit Channel Isolation Measurements

**FIGURE 5:**
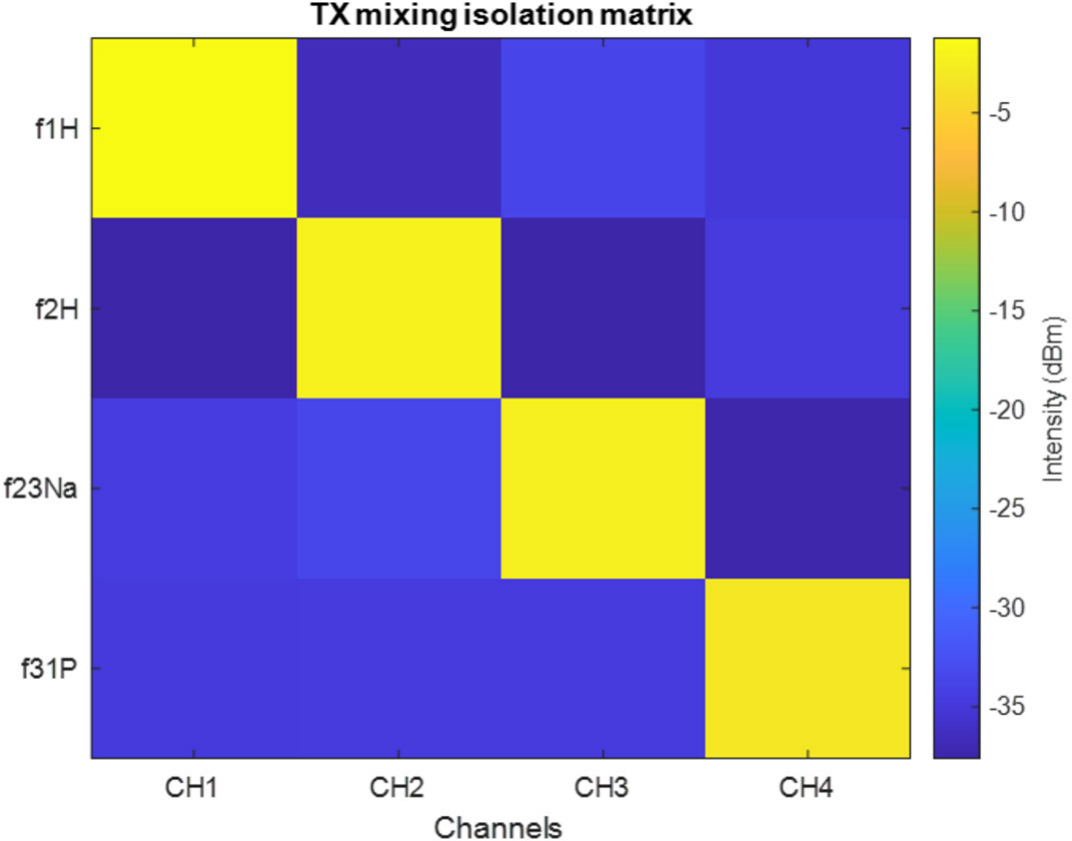
Measured isolation matrix for the four transmit mixing channels when simultaneously transmitting at four different frequencies. All the measurements were normalized to the maximum intensity.

The isolation matrix of the transmit mixing channels with all measurements normalized to the maximum intensity is shown in Figure 5. The results show excellent isolation as each mixing channel generated the desired Lamor frequencies accurately, while the nondesired frequency components from the other channels were at least 30 dB lower.

#### 3.1.2 Receive Channel Isolation Measurements

**FIGURE 6:**
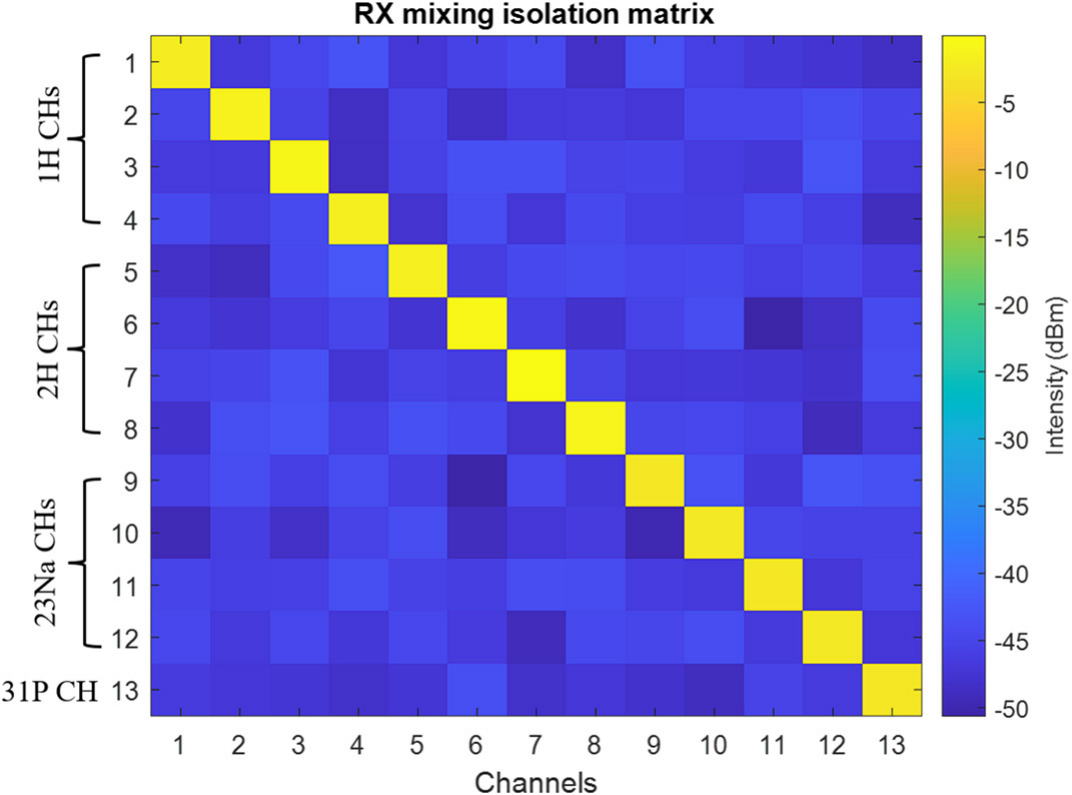
Measured isolation matrix for the 13 receive mixing channels when simultaneously receiving at four different frequencies. All the measurements were normalized to the maximum intensity.

The receive channel isolation matrix was also normalized to the maximum intensity and is shown in Figure 6. The isolation matrix shows that all the receive mixing channels successfully converted the different Larmor frequencies to the 20.3‐MHz IF frequency, and at least −30 dB in‐band or out‐of‐band isolation was observed on any other channels.

#### 3.1.3 Transmit Pulse Envelope Measurement

**FIGURE 7:**
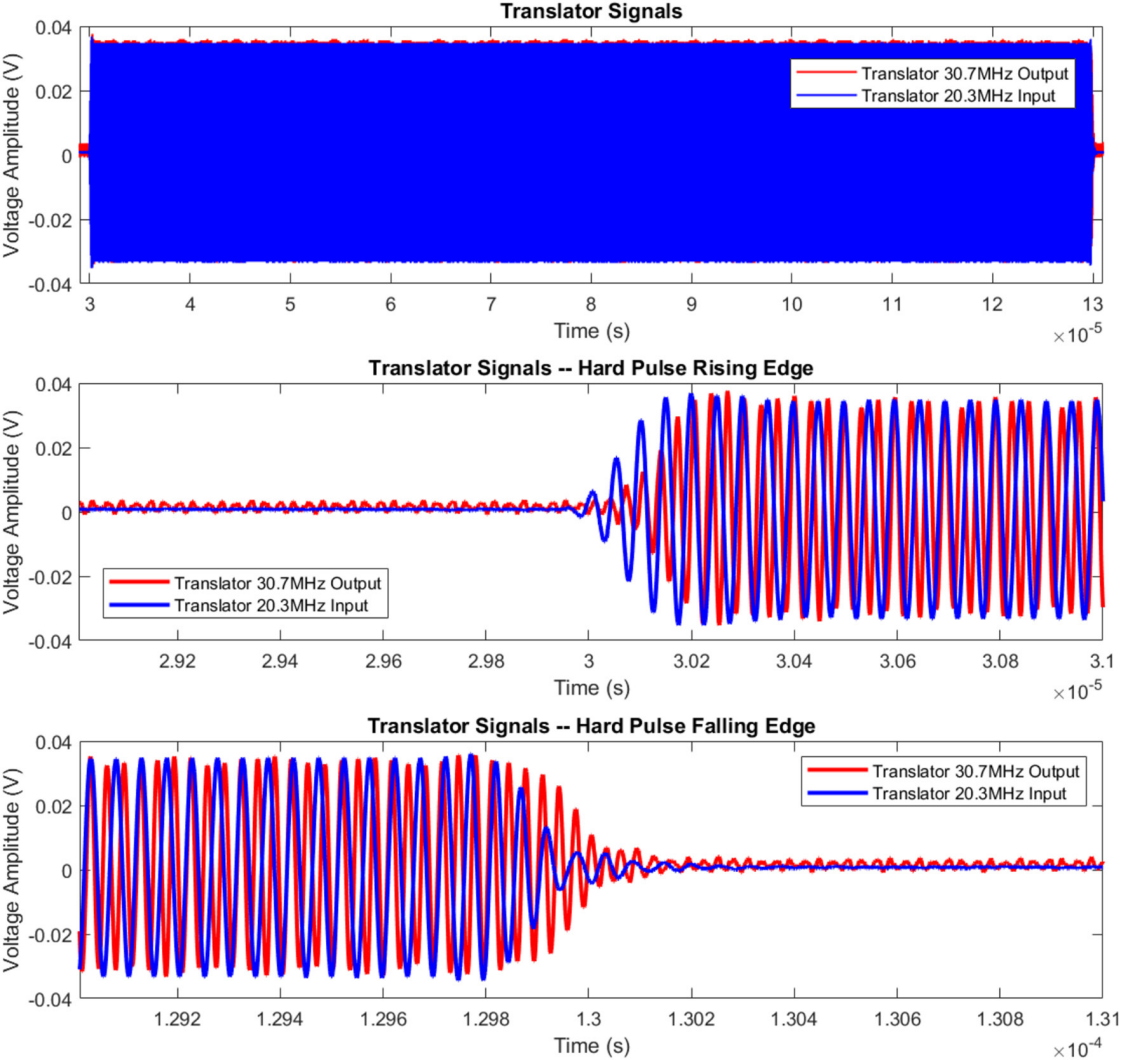
Oscilloscope measurements of the transmit mixing channel input (20.3 MHz, blue) and output (30.7 MHz, red) waveforms for a hard pulse at the ^2^H frequency. The two waveforms were captured separately, both triggered off the same TX gate signal, with gains adjusted to allow direct visual comparison. (Top) Full pulse waveform. (Middle) Zoomed‐in view of the rising edge. (Bottom) Zoomed‐in view of the falling edge. The pulse envelope is well preserved after frequency translation, with a group delay of approximately 70 ns observed at the output, consistent with the BBP‐30 + bandpass filter datasheet specification of 62 ns.

The input (20.3 MHz) and output (30.7 MHz) waveforms of the transmit mixing channel are shown in Figure 7. The results confirm that the pulse envelope is well preserved after frequency translation. A small group delay of approximately 70 ns is observed at the output, which is consistent with the typical group delay of approximately 62 ns specified in the BBP‐30 + bandpass filter (Mini‐Circuits) datasheet at this frequency and is accounted for in the instrumentation delay of the pulse sequence. The slightly elevated noise floor at the output during the off period is expected from the mixer and gain stage noise contribution and has no practical impact on system performance.

### 3.2 MR Experiment Results

#### 3.2.1 Simultaneous ^1^H, ^2^H, and ^23^Na FID Experiments

**FIGURE 8:**
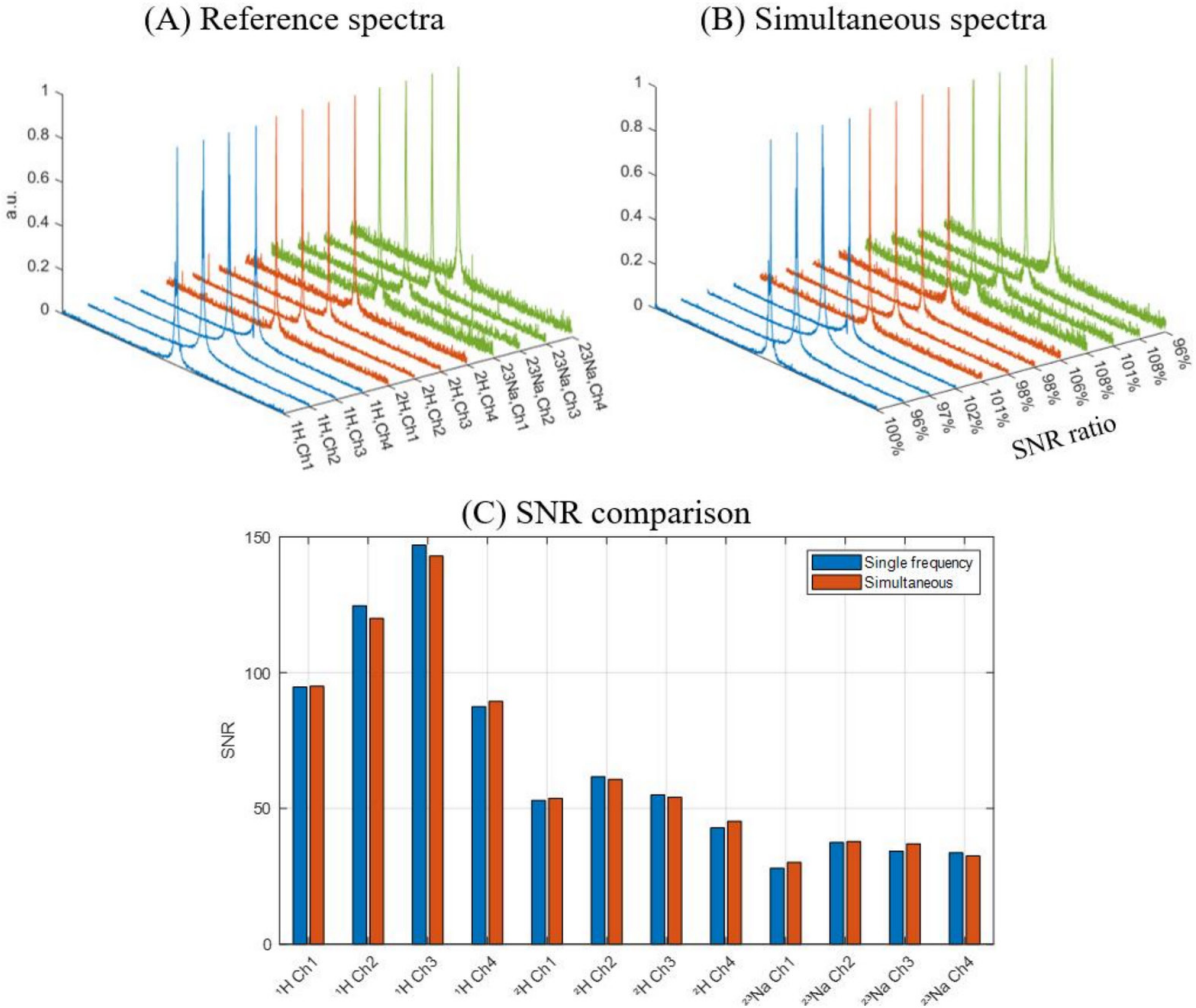
^1^H, ^2^H, and ^23^Na FID results with 24 averages. (A) Reference spectra acquired with single frequency operation. (B) Spectra acquired with interleaved transmission and simultaneous receive operation at three frequencies, with the SNR ratio of simultaneous to reference labeled on the axis for each channel and frequency. (C) Grouped bar histogram comparing the absolute SNR values between single frequency (blue) and simultaneous (orange) operation for all coil channels at each frequency. No significant SNR penalty was observed when performing the simultaneous acquisition.

The spectra from basic FID experiments performed at ^1^H, ^2^H, and ^23^Na are shown for SNR comparisons in Figure 8. The SNR was quantified by dividing the integration of 30 points around the signal peak over the standard deviation of the first 300 points in the spectra. The results indicate that the three‐frequency signals were successfully acquired simultaneously from all four receive array coils, and the total scan time was reduced by 1/3, while maintaining a comparable SNR performance to the single frequency acquisition.

#### 3.2.2 Simultaneous ^2^H and ^23^Na GRE Imaging

**FIGURE 9:**
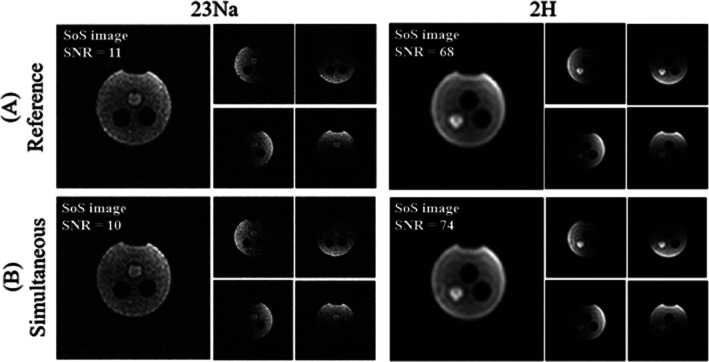
^23^Na and ^2^H GRE array images with 64 averages acquired (A) individually with single frequency operation and (B) simultaneous transmit and receive operation with both frequencies. The ^2^H images were acquired with the same gradient parameters as ^23^Na, resulting in an effective FOV of 275.7 mm and slice thickness of 34.5 mm, and were interpolated to 256 × 256 and cropped to the central 149 × 149 pixels to match the ^23^Na FOV of 160 mm. The SNR is comparable between the simultaneous and reference scans for both frequencies.

GRE imaging results are shown in Figure 9. The ^23^Na images were acquired with a 20‐mm slice, 160‐mm FOV, and 64*64 matrix size and later interpolated to 256*256 in postprocessing. The ^2^H acquisition used the same ^23^Na gradient parameters. Due to the difference in gyromagnetic ratios (γ(^23^Na) = 11.262 MHz/T, γ(^2^H) = 6.536 MHz/T, ratio ≈ 1.72), the same gradient strength resulted in an effective FOV of 275.7 mm and a slice thickness of 34.5 mm for ^2^H. To present the images at the same effective FOV as ^23^Na, the ^2^H images were first interpolated to 256 × 256, and then the central 149 × 149 pixels were cropped to match the ^23^Na FOV of 160 mm. The parallel imaging results from each channel were combined using the sum‐of‐squares method. The SNR was quantified by dividing the mean value in the signal region by the standard deviation of the noise region, where the signal region was defined by the points with 35% and above values of the maximum and the noise region was chosen as a 64 × 64 box on the top left corner. As shown in the results, the images acquired in both cases were almost identical and the total scan time was reduced by half. The slight SNR variation was likely attributed to RF calibration differences between the two experiments, as the calibration accuracy was limited by the low SNR X‐nuclear experiments.

#### 3.2.3 Simultaneous Water‐Suppressed ^1^H and ^31^P Spectroscopy

**FIGURE 10:**
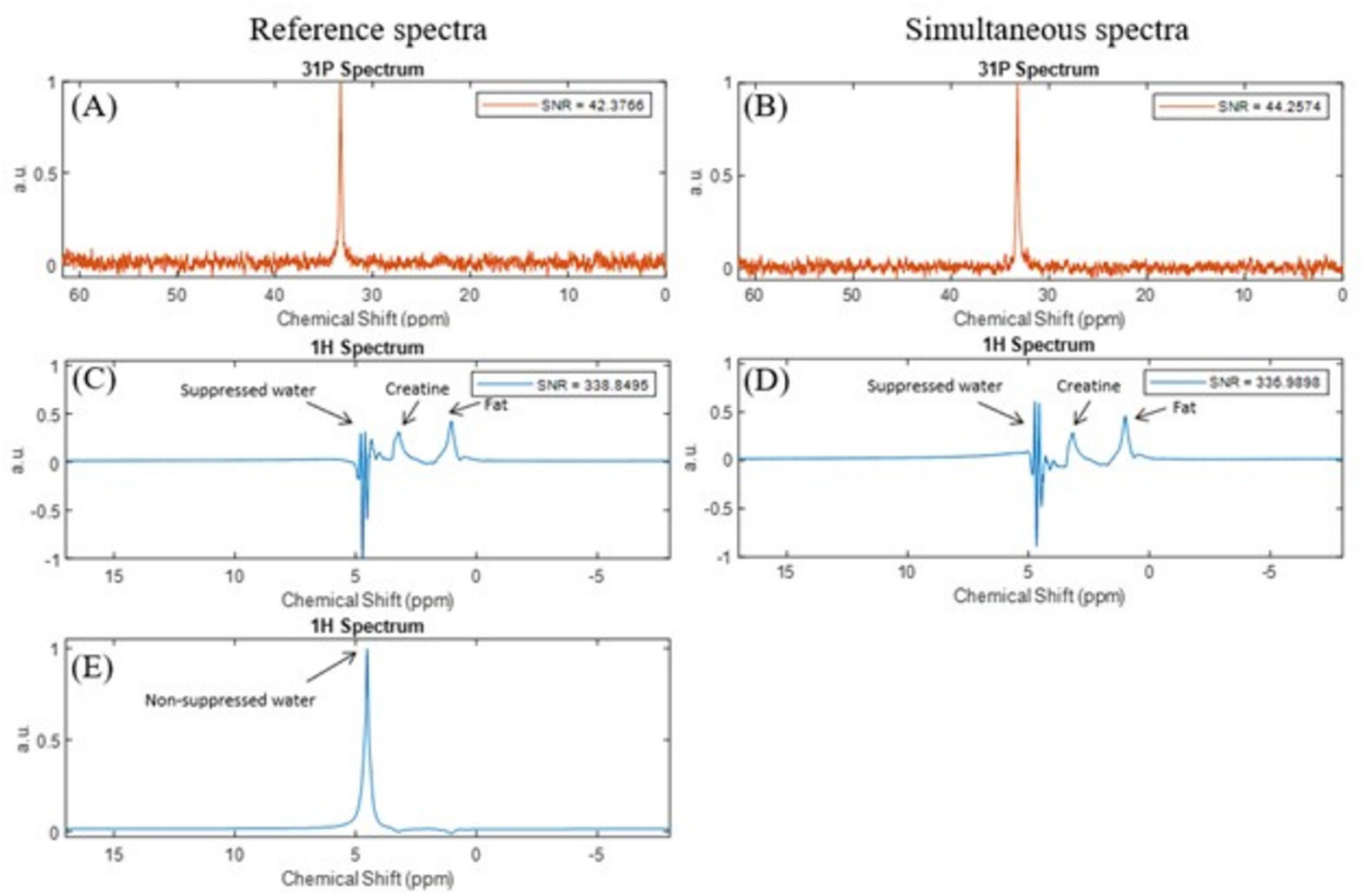
Water‐suppressed ^1^H and ^31^P spectroscopy experiment results acquired with 100 averages. Reference (A) ^31^P and (C) ^1^H spectra were acquired in single frequency mode and compared to the simultaneously acquired (B) ^31^P and (D) ^1^H spectra. The simultaneously acquired spectra show comparable SNR. A separately acquired nonsuppressed ^1^H spectrum (E) was plotted to demonstrate water suppression.

The reference and simultaneous ^1^H and ^31^P spectra, as well as a separately acquired nonsuppressed ^1^H spectrum, were shown in Figure 10. All spectra were plotted using the real component after zero‐order phase correction. Line broadening of 3 Hz was applied to all the ^31^P spectra for visualization purposes. The SNR was quantified by using the signal divided by the standard deviation of the first 400 points in the noise floor. The signal was defined as the peak signal for the ^31^P spectra and integration of both the creatine and fat signal for the ^1^H spectra. The results demonstrate that water suppression was successfully performed for both operation modes, and the simultaneously acquired spectra saved half of the scan time while maintaining comparable SNR performance as the reference spectra.

## 4 Discussion and Conclusions

To modify a scanner for simultaneous or interleaved multinuclear experiments, the decoupling channel is often used to generate the second nucleus (usually ^1^H) and the receiver frequency is altered by providing an external LO signal. However, this approach results in two issues: (1) The transmit frequency is usually limited to two nuclei at a time, and (2) the received signal usually contains a phase shift that needs to be corrected in postprocessing. Both issues are addressed in the add‐on system described here.

One of the advantages of the proposed system is its flexibility to adapt to different frequencies. Due to the multichannel design of the mixing and LO channels, the number of nuclei supported is not limited by the available transmit channels on the system. In the current implementation, up to four nuclei can be examined in one experiment, limited by the number of LO channels on a single ad9959 board and the number of transmit mixing channels installed. This can be readily scaled by adding more ad9959 boards synchronized to the same 10‐MHz reference clock and additional transmit mixing channels, with the practical upper limit set by the number of available spectrometer receiver channels. Simply by changing the LO frequencies and replacing the band‐pass filters, it can also support experiments at different field strengths.

Both simultaneous and interleaved acquisition methods have the benefit of reducing overall scan time in a multinuclear experiment, but one approach might be more preferable than the other depending on the needs of the study. Simultaneous acquisition of multiple nuclei could provide time‐resolved information for all the nuclei. However, given the intrinsic differences between nuclei, such as gyromagnetic ratios, T1 and T2 values, and sensitivities, it might be beneficial to interleave the different nuclei in the same TR and optimize scan parameters independently.

As demonstrated by the three experiments in this paper, simultaneous or interleaved transmit approaches can be freely chosen. Different pulse sequence modifications would be needed in different scenarios to generate multiple transmit pulses and to provide additional gating signals. On the receive side, although only the simultaneous acquisition approach is demonstrated, the proposed system can inherently support interleaved acquisition with pulse sequence modification to turn on specific receiver channels at certain times.

The proposed system utilizes multiple transmit and receive mixing paths to support simultaneous multinuclear experiments. Instead of solely adjusting the receive frequency by inserting an external LO signal, the transmit and receive frequencies are both converted by the same LO, and therefore, the phase is always stable. This eliminates the need for phase correction in postprocessing. The transmit pulse envelope measurement further confirms that the frequency translation process preserves the RF pulse shape, with the small observed group delay being a fixed and reproducible consequence of the bandpass filter phase response and already accounted for in the pulse sequence. Especially for a proton‐only system, the proposed system can be implemented with an additional broadband RFPA to enable not only the X‐nuclear capability but also the true flexible simultaneous multinuclear function.

This system was installed and tested in a research laboratory environment that mainly focuses on phantom studies to assess coil and hardware development. It is technically straightforward to retrofit a clinical scanner in a manner similar to work that has been presented using frequency translation [35, 36]. A nontrivial consideration, however, is the special care that would need to be taken to ensure RF safety. The clinical system monitors the transmitted RF power to prevent excessive SAR in the subject, but the calibration of the safety monitoring system could be affected by the modification on the transmit side. If the add‐on system is to be installed on a clinical system, additional power monitoring hardware may be needed after the transmit mixing hardware. Additional RF power calculations at each frequency of interest also would need to be performed and verified to not exceed the SAR limit. It is also important to note that in simultaneous or rapidly interleaved multinuclear transmit scenarios, the SAR contributions from each frequency should not be assessed independently. The local SAR hotspot distributions can differ across frequencies, and their combined effect on tissue heating can be more complex than a simple superposition of single‐frequency SAR models. Therefore, multifrequency electromagnetic simulations will be needed to accurately evaluate the SAR contributed from all frequencies.

We have demonstrated the performance of the system on phantoms with physiologically relevant concentrations of ^1^H and ^31^P metabolites. We successfully acquired simultaneous water‐suppressed ^1^H and ^31^P spectra with comparable SNR to single frequency operation. It should be noted that the SNR comparison in this work was performed with the add‐on system in place for both the reference and simultaneous acquisitions and therefore does not directly quantify the insertion loss of the system itself. However, as reported in other frequency translation systems [26, 35, 36], when sufficient gain is applied prior to the mixing stage, the noise contribution of the mixer is suppressed and SNR degradation is minimal. These results indicate the feasibility of immediate implementation of the system on the study of ex vivo skeletal muscle samples. In the future, the implementation of this system on preclinical studies could be explored after the previously discussed considerations have been addressed.

To conclude, we demonstrated a multinuclear system for retrofitting conventional MRI systems with true simultaneous and/or interleaved multinuclear acquisition. This was implemented on an EVO spectrometer at 4.7 T and can be readily adopted by other research groups using preclinical MR systems with similar architecture. Translation to clinical MR systems is also feasible in principle but would require additional consideration of manufacturer liability, regulatory requirements, and RF safety validation, as discussed earlier. Simultaneous and interleaved excitation and simultaneous acquisition of multiple nuclei were successfully demonstrated, and no SNR loss was observed when compared to the single frequency operation. This proposed multinuclear system utilizes transmit and receive mixing hardware that was developed in our lab and a commercially available DDS board, with a total component cost estimated to be under $15,000. While a broadband RFPA is additionally required for implementation on a proton‐only system with cost varying depending on specifications, this is still much lower than commercial X‐nuclear upgrade packages that can cost up to $500,000 [33] and potentially could promote multinuclear clinical and research studies.

## Author Contributions

Jue Hou and Steven M. Wright contributed to the conceptualization and hardware design of the system. Jue Hou developed and assembled the system prototype, conducted the experiments with assistance from Courtney Bauer, and wrote the manuscript. Jue Hou and Courtney Bauer processed the data. Edith Touchet‐Valle designed and built the ^1^H/^31^P coils and prepared the ^1^H/^31^P metabolite phantom. Mary P. McDougall and Steven M. Wright acquired funding for this project. Steven M. Wright supervised the study. All authors reviewed and revised the manuscript and approved the final version for submission.

## Funding

Research reported in this publication was supported by the National Institute of Biomedical Imaging and Bioengineering of the National Institutes of Health under award number R01EB028533.

## Conflicts of Interest

The authors declare no conflicts of interest.

## Data Availability

The data and design files supporting the findings of this study are available from the corresponding author upon reasonable request.
